# Effects of the Labor Inspection Authority’s regulatory tools on physician-certified sick leave and employee health in Norwegian home-care services – a cluster randomized controlled trial

**DOI:** 10.5271/sjweh.4126

**Published:** 2024-01-01

**Authors:** Bjørnar Finnanger Garshol, Stein Knardahl, Jan Shahid Emberland, Øivind Skare, Håkon A Johannessen

**Affiliations:** 1 Department of Work Psychology and Physiology, National Institute of Occupational Health, Oslo, Norway.; 2 National Institute of Occupational Health, Oslo, Norway.; 3 Department of Occupational Medicine and Epidemiology, National Institute of Occupational Health, Oslo, Norway.

**Keywords:** labor inspection, occupational health, occupational safety, OSH enforcement, OSH intervention, self-reported health, sickness absence

## Abstract

**Objective:**

This study aimed to determine the effects of the Labor Inspection Authority’s regulatory tools on physician-certified sick leave and self-reported health outcomes among employees in municipal home-care services in Norway.

**Methods:**

We conducted a cluster-randomized controlled trial in the home-care service sector, and 96 eligible municipalities were randomly assigned to one of three groups: (i) labor inspection visits, based on the Labor Inspection Authority’s standard inspections; (ii) guidance-through-workshops, where participants from home-care services met with labor inspectors to receive information and discuss relevant topics; and (iii) the control group. Data on employee self-reported health (N=1669) were collected at baseline and 6 and 12 months after the interventions. Additionally, registry data (N=1202) on diagnosis specific physician-certified sick leave were collected for 18 months after the interventions.

**Results:**

We found no statistically significant effects of either intervention on self-reported health outcomes. There was, for both interventions, a pattern of decrease in days and periods of physician-certified sick leave due to musculoskeletal diagnoses and increase in days and periods of physician-certified sick leave due to psychological diagnoses, but these were not statistically significant.

**Conclusion:**

Labor inspections and guidance-through-workshops had no statistically significant effect on self-reported health and physician-certified sick leave. The results should be interpreted with caution given the low response rate and subsequent attrition, and in the context of the COVID-19 pandemic. Future studies, in various industries, should further elucidate whether regulatory tools influence employee health and sick leave due to musculoskeletal and mental disorders.

Work-related ill-health and sickness absence incur large personal and societal costs (1). Risk factors in the work environment, such as mechanical and psychosocial factors, have been linked to musculoskeletal complaints (2, 3), mental distress (4, 5), and subsequent sickness absence (6–9). In Norway, an estimated 40% of lower back pain cases can be attributed to mechanical and psychosocial work factors (10), while 25% of mental distress cases may be attributed to psychosocial factors (11). Some sectors have a higher-than-average prevalence of sickness absence, such as the home-care services sector, where nurses have a sickness absence rate of 11% in Norway, compared to the national average of 5.8% (12). There is a high prevalence of musculoskeletal and mental disorders among home-care employees (13, 14), with the work environment being characterized by both job strain (15), such as high work intensity and emotional demands (16) and strenuous work tasks (17), for example awkward postures and lifting/supporting patients. The sector has also been facing increasing demands due to an increase in the elderly population together with increased restructuring to focus on providing care at home instead of in long-term care institutions, both of which could affect working conditions at the services (18).

The enforcement of occupational safety and health (OSH) laws and regulations is essential to protect employee health and ensure a good working environment (19, 20). In Norway, the Working Environment Act and Internal Control Regulation set standards to which organizations are obliged to adhere. These legislative and regulatory measures are enforced by the Norwegian Labor Inspection Authority (NLIA), with labor inspections being their main regulatory tool. The NLIA also provides guidance to organizations on how to understand relevant laws and regulations and on potential risk factors and their health impact, both in conjunction with the inspections themselves and as a separate activity through seminars and workshops.

Previous research on the effects of regulatory measures on OSH noted that labor inspections increase compliance with regulations and reduces the incidence of injuries (21–23). However, most research has been conducted in the manufacturing and construction sectors, and there is little knowledge of potential effects in the healthcare sectors (21, 22). Furthermore, limited research has been conducted on the effect of regulatory measures on psychological and musculoskeletal disorders and sickness absence (22).

Consequently, this study aimed to determine the effects of labor inspections and guidance workshops on self-reported health complaints and physician-certified sick leave due to musculoskeletal and psychological diagnoses of employees in home-care services. Based on previous studies on the effects on compliance and injuries, we assumed that regulatory tools could influence both physician-certified sick leave and self-reported health.

## Methods

### Design

The present study was a cluster-randomized controlled trial based on a probability sample of home-care service workers in Norway. A cluster-randomized design was chosen as the work environment of home-care services are inherently clusters. The study consisted of two intervention groups, labor inspections and guidance workshops, and one control group. This study is part of a larger project – the Effects of the Labor Inspection Authority’s Regulatory Tools on Work Environment and Health in the Norwegian Home-care Services project (EAVH project) – Clinical Trials ID: NCT0355163 (Registered 26 February 2019), and a full description of the project can be found in the published protocol (24).

### Recruitment and participants

In January 2019, Norway had 422 municipalities with home-care services varying in size from 3–>4000 employees (24) For this study, eligible municipalities were those where home-care services employed >20–<100 care workers. This range was chosen to reduce the intra-cluster variability, thereby reducing the required sample size. Additionally, a majority of the home-care services in Norway at the time fell within this range (24). Ineligible municipalities were those that fell outside this scope or had recently undergone labor inspections, that is in 2017–2018. Based on sample size calculations (24), 132 of the 187 eligible municipalities were randomly assigned to one of the four original study groups. The project lead conducted randomization using random numbers assigned to each municipality, sorting, and then assigning the first 33 to one group, the next 33 to another, and so on. We then informed the municipalities about the planned study through letters and email and invited them to participate. Participating municipalities were asked to provide a contact person from the municipality’s home-care services, who provided overviews of the current employees, including contact information, such as phone numbers and email addresses. This information was subsequently used to invite all the employees to participate in the study.

**Figure 1:**
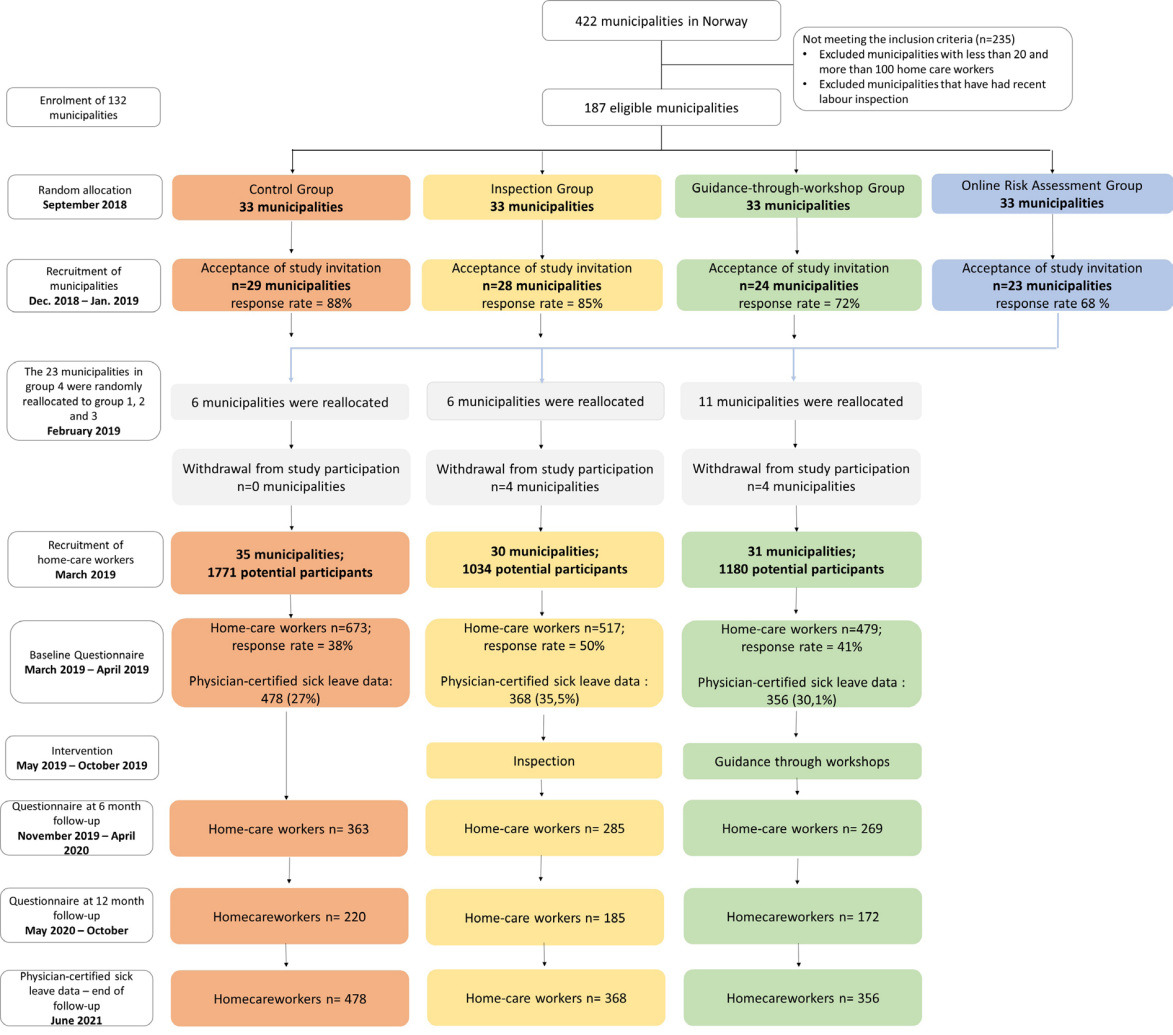
Flowchart illustrating the process of cluster allocation, intervention implementation and data collection for the project, including time points for implementation and the endpoint for registry data.

Overall, 104 of the 132 randomly assigned municipalities were recruited before the planned implementation of the interventions. Originally three intervention groups were planned (24), but – due to fewer recruited municipalities than expected – those in the last intervention group (online risk assessment) were randomly reallocated to the remaining two interventions and the control group [see Finnanger Garshol et al (25) for further details]. In total, 96 municipalities participated in the study, and these had 3985 employees in their home-care services. Out of these 3985 potential participants, there were 673 respondents from 35 municipalities in the control group at baseline, 517 from 30 municipalities in the inspection intervention group and 479 from 31 municipalities in the guidance intervention group. In total, we had 1669 respondents at baseline, giving a response rate of 41.9%. Of these, 1202 respondents consented to the collection of registry data: 478, 368, and 356 from the control, inspection, and guidance groups, respectively. There were no drop-out in the registry data, while the overall drop-out rate among those who responded at baseline was 65.2% over the course of the study. Those who stopped responding were younger, had less education, had a lower percentage of full-time equivalent employment, and were more often listed as “other care staff” (25). Figure 1, adapted from the project protocol (24), provides an overview of the study recruitment and the flow of participants including endpoint for registry data.

### Interventions

This study included two interventions, labor inspections and guidance workshops, and a control group. Both interventions were implemented between May and October 2019.

### Labor inspections

The inspection intervention was structured according to the NLIA’s standardized inspection routines. Participating workplaces in the municipality received notice and information on impending inspections three weeks in advance. The inspections were carried out by trained inspectors at the home-care service offices. Individual care recipients’ homes were not included in the inspections. A standardized checklist, operationalizing relevant legislation (the Internal Control Regulation and the Working Environment Act) was used during the inspections. The checklist was used to check compliance with the legislation and focused on exposures related to the psychosocial, organizational, and mechanical work environment. In addition, inspectors also provided information and guidance on how to comply with labor regulations. Post-inspection, reports were made specifying areas of non-compliance at each home-care service, and the actions they should take to avoid sanctions and fines.

### Guidance-through-workshop

Based on geographical location, 5–7 home-care services were assigned to one-time workshops, which two trained labor inspectors from the NLIA led. The manager, safety representative, and employee representatives from each participating home-care service were invited to the workshop and informed that the topic was ‘work environment and employee health’. These representatives were also asked in advance to prepare presentations on specific challenges employees in their own working environments face. The attending inspectors were instructed to provide advice to the participants on these concerns, based on relevant OSH legislation and regulations.

### Control group

The control group received ‘care as usual’, meaning that no inspections or guidance-workshops were undertaken. The control group completed the same work environment and health questionnaires as the inspection and guidance groups.

### Data collection

Data were collected using a web-based questionnaire developed by the National Institute of Occupational Health (STAMI) in Norway. It could be completed in multiple sessions and each participant received a unique sign-in code. A paper-based version was provided upon request. We collected data prior to the interventions (baseline), and at 6 and 12 months after the interventions for all three groups.

### Participants demographics

We collected demographic information from each participant, such as age, gender, marital status, occupation, level of completed education and their percentage employment, that is the full-time equivalent (FTE) percentage based on what is considered a standard full-time position (about 37.5 hours a week), and occupation based on the Norwegian version of the International Standard Classification of Occupations 2008.

### Outcome variables

Subjective general health was assessed using a single-item question, ‘How would you rate your health in general?’. Responses were given in the following categories 0=very bad, 1=bad, 2=moderate, 3=good, and 4=very good. We measured one domain of mental health, mental distress, while we focused on musculoskeletal complaints and pain for physical health.

Mental distress, defined as symptoms of anxiety and depression, was measured using the five-item version of the Hopkins Symptom Checklist (HSCL-5) (26). Each item was rated from 1 (not at all) to 4 (extremely) and based on symptoms experienced in the previous week. The HSCL-5 is a reliable and validated instrument that performs similarly to more expansive versions, HSCL-10 and 25 (26).

Musculoskeletal complaints were measured using six items adapted from Steingrimsdottir et al (27). The six separate items asked the participants to rate if they in the last four weeks had been troubled by (i) headaches; (ii) neck pain; (iii) back pain; (iv) pain in the shoulder or upper arm; (v) pain in the lower arm, wrist, or hands; or (vi) pain in the hips, legs, knees, or feet during the last four weeks. Each item had the following response categories: 1=not troubled, 2=a little troubled, 3=intensely troubled and 4=very intensely troubled. In addition, the participants were asked to assess their *general pain intensity in the preceding week* using an 11-point numerical rating scale ranging from 0 (no pain) to 10 (worst possible pain). Such numerical rating scales have previously been shown to be applicable across settings, with higher compliance and ease of use than other unidimensional pain measures (28)

Registry data on physician-certified sick leave were obtained for the period 1 January 2018 to 30 June 2021 from the Norwegian Labor and Welfare Administration. This included the start and end date for all physician-certified sick leaves for the period, along with the accompanying diagnoses based on the International Classification of Primary Care 2 (ICPC-2). These diagnoses were recoded into different categories: (i) all musculoskeletal and psychological diagnoses (L and P-codes), (ii) all musculoskeletal diagnoses (L-codes), and (iii) all psychological diagnoses (P-codes). These were chosen as diagnoses of interest as they are potentially caused by psychosocial and mechanical risk factors in the work environment (2–9). Using these categories, we created variables for the total number of days of sick leave, where we counted and added together all days of sick leave due to the diagnoses of interest for 18 months post-interventions. We also created variables for number of sick leave periods, that is the total number of sick leaves due to the diagnoses of interest for 18 months post-interventions. Corresponding days and sick leaves due to L and/or P diagnoses in the 12 months preceding the interventions were used as a measure of baseline sickness absence. The 18 months post-intervention and 12 months pre-intervention periods were calculated for each individual based on when the intervention or guidance workshop had been conducted for their service or when the baseline questionnaire had been disseminated for the control group.

### Statistical analyses

All analyses were performed using STATA (version 16.1, Stata Corp, College Station, TX, USA). T-tests were conducted to compare the demographic variables of the two intervention groups with those of the control-group at baseline. Changes in self-reported health outcomes were analyzed separately for each outcome using linear mixed models with participants nested within the municipalities as random effects. The models included time, time × group and employment percentage as independent variables. The time variable was based on the different rounds of data collection, that is first round as time=1, etc. The FTE was included as there was a difference between the guidance and control group at baseline. The variable was viewed as intrinsically linked with exposure as it is a measure of how much time an employee spends at work and thus is exposed to the work environment. Tests of the sick leave data showed overdispersion, meaning an assumption of a Poisson distribution was not appropriate. Thus, we used mixed negative binomial regression to analyze physician-certified sick leave, with municipalities included as random effects. All participants were analyzed based on intention-to-treat. The analyses were adjusted for outcome variables at baseline as recommended for randomized controlled trials (29). Further, baseline adjustment was also used to address a group difference at baseline on certified sick leave due to musculoskeletal and psychological diagnoses. To account for multiple testing of self-reported measures, the Benjamini-Hochberg test was used to provide adjusted P-values (30). The level of significance was set at P<0.05.

### Ethics

This study was conducted in accordance with the principles of the Declaration of Helsinki (31). All participants provided written informed consent and were informed of their right to withdraw from the study at any time. While the participating services received no incentives or compensation for participation, individual participants could win a 15 000 Norwegian krone gift certificate. The study was assessed by the Regional Committees for Medical and Health Research Ethics, and the handling of personal data and data storage was approved by the Norwegian Centre for Data Research (Nr: 566128). The project stored all self-reported data electronically and the data were kept separate from any identifying information.

## Results

There were no statistically significant group differences on the demographics age, gender, marital status, educational background, type of employment or leadership responsibilities (table 1). However, there was a difference in mean percentage employment, as the guidance-groups mean percentage employment was 3.0 percentage points lower than that of the control group. Of the 1669 respondents, 467 did not consent to the collection of registry data. There was no statistically significant difference in baseline self-reported health between those who consented and those who did not. Those who did not consent were on average 2.4 years younger (P<0.001), had 0.1 years less education (P= 0.03) and 2.8 percentage points less FTE employment (P=0.02) than those who consented. Among the participants who consented (N=1202), there were no statistically significant demographic between-group differences, except for, as the main sample, a difference in mean percentage employment. There was a statistically significant group difference at baseline on physician-certified sick leave due to musculoskeletal and psychological diagnoses, with a higher proportion of participants with sick leave in the control (27.6%) versus inspection (18.2%) and guidance (21.1%) groups (table 2).

**Table 1:** Characteristics of study participants. [SD=standard deviation.]

		Inspection (N= 517)			Guidance-through-workshop (N= 479)			Control (N= 673)	
		N (%)	Mean (SD)		N (%)	Mean (SD)		N (%)	Mean (SD)
Gender									
	Male	22 (4.3)			22 (4.6)			28 (4.2)	
	Female	495 (95.7)			457 (95.4)			645 (95.8)	
Age			46.1 (11.6)			44.7 (12.2)			45.3 (12.1)
Marital status									
	Unmarried	70 (13.7)			64 (13.5)			98 (14.9)	
	Married/ cohabiting	398 (77.7)			362 (76.7)			508 (77.2)	
	Widow/widower	12 (2.3)			7 (1.5)			7 (1.1)	
	Divorced/ separated	32 (6.3)			39 (8.3)			45 (6.8)	
Education level									
	Primary school	25 (5.1)			16 (3.5)			18 (2.8)	
	High school	241 (48.8)			239 (51.9)			312 (48.7)	
	University/college ≤3 years	209 (42.3)			190 (41.3)			289 (45.1)	
	University/college >3 years	19 (3.8)			15 (3.3)			22 (3.4)	
Type of employment									
	Permanent	482 (94.1)			434 (92.7)			619 (93.5)	
	Temporary	16 (3.1)			16 (3.4)			18 (2.7)	
	Substitute/on-call	11 (2.2)			16 (3.4)			23 (3.5)	
	Other	3 (0.6)			2 (0.4)			2 (0.3)	
Percentage of full-time equivalent employment			79.0 (22.1)			78.3 (22.6) ^a^			81.2 (21.5)
Leader responsibilities									
	Top tier leader	89 (17.7)			92 (19.7)			106 (16.3)	
	Middle tier leader	44 (8.8)			37 (7.9)			48 (7.4)	
	No leadership responsibilities	370 (73.6)			337 (72.3)			496 (76.3)	
Consented to use of registry data on physician-certified sick leave		368 (71.2)			356 (74.3)			478 (71.0)	

^a^ Significantly different from controls, P≤0.05

**Table 2:** Sick leave data for the three different groups pre-intervention and post-intervention. [SD=standard deviation.]

		Inspection (N=368)				Guidance (N=356)				Control (N=478)		
		N (%)	Δ%	Mean (SD)		N (%)	Δ%	Mean (SD)		N (%)	Δ%	Mean (SD)
Participants with one or more physician-certified sick leave periods pre-intervention ^b^												
	Musculoskeletal and psychological diagnoses	67 (18.2^) a^				75 (21.1) a				132 (27.6)		
	Musculoskeletal diagnoses	46 (12.5) a				60 (16.9)				99 (20.7)		
	Psychological diagnoses	28 (7.6)				20 (5.6)				41 (8.6)		
Participants with one or more physician-certified sick leave periods post-intervention ^c^												
	Musculoskeletal and psychological diagnoses	94 (25.5) a				109 (30.6)				154 (32.2)		
	Musculoskeletal diagnoses	72 (19.6)				82 (23.0)				118 (24.7)		
	Psychological diagnoses	33 (9.0)				36 (10.1)				46 (9.6)		
Overall change in percentage-points from pre- to post-intervention for musculoskeletal and psychological diagnoses			7.3				9.6				4.6	
Physician-certified sick leave periods pre-intervention ^a^												
	Musculoskeletal and psychological diagnoses			0.27 (0.7) a				0.29 (0.6) a				0.38 (0.7)
	Musculoskeletal diagnoses			0.18 (0.6) a				0.21 (0.5)				0.28 (0.7)
	Psychological diagnoses			0.09 (0.4)				0.07 (0.3)				0.10 (0.3)
Physician-certified sick leave periods post-intervention ^c^												
	Musculoskeletal and psychological diagnoses			0.45 (1.0)				0.48 (0.9)				0.47 (0.8)
	Musculoskeletal diagnoses			0.31 (0.7)				0.33 (0.7)				0.35 (0.7)
	Psychological diagnoses			0.14 (0.6)				0.14 (0.5)				0.11 (0.4)
Physician-certified sick leave days pre-intervention ^b^												
	Musculoskeletal and psychological diagnoses			11.90 (43.6) a				16.08 (64.6) a				26.22 (74.0)
	Musculoskeletal diagnoses			9.05 (41.7) a				12.25 (55.4) a				19.82 (66.5)
	Psychological diagnoses			2.86 (13.8)				4.82 (34.5)				6.39 (35.3)
Physician-certified sick leave days post-intervention ^c^												
	Musculoskeletal and psychological diagnoses			30.75 (82.1)				32.11 (84.1)				34.52 (86.2)
	Musculoskeletal diagnoses			20.58 (67.1)				21.84 (68.6)				23.57 (75.2)
	Psychological diagnoses			10.17 (50.6)				10.27 (51.2)				10.94 (46.8)

^a^ Difference from controls, P<0.05. ^b^ 12-month period pre-intervention. ^c^ 18-month period post-intervention..

There were no statistically significant effects of either intervention on the self-reported employee health outcomes (table 3), except for an initial negative effect of the inspection intervention on subjective general health at 12 months prior to adjusting for multiple testing. After adjusting the P-values using the Benjamini-Hochberg test, this effect was no longer observed.

**Table 3:** Linear mixed models with baseline measures comparing the interventions groups with the control group to assess their effect on health outcomes at 6- and 12-months post-interventions with baseline measures for all three groups. [ICC=interclass correlation coefficient; SD=standard deviation; Coef=coefficient; CI=confidence intervals.]

	Baseline						First follow-up						Second follow-up					ICC ^a^
	Inspection		Guidance		Control		Inspection			Guidance			Inspection			Guidance		
	Mean (SD)		Mean (SD)		Mean (SD)		Coef.	95% CI		Coef.	95% CI		Coef.	95% CI		Coef.	95% CI	
General health (0–4) ^b^	2.09 (0.82)		2.10 (0.81)		2.09 (0.82)		-0.06	-0.18–0.05		-0.10	-0.22–0.01		-0.15	-0.29– -0.01		-0.12	-0.26–0.02	0.019
Mental distress (1–4)	1.35 (0.51)		1.38 (0.51)		1.43 (0.54)		-0.07	-0.15–0.01		-0.07	-0.15–0.01		0.03	-0.06–0.12		-0.02	-0.12–0.07	<0.001
General pain (0–10)	3.18 (2.35)		3.28 (2.41)		3.30 (2.34)		-0.01	-0.36–0.34		-0.14	-0.49–0.20		-0.20	-0.62–0.22		-0.01	-0.42–0.42	0.021
Headache (1–4)	1.83 (0.88)		1.89 (0.81)		1.85 (0.85)		0.04	-0.08–0.16		-0.01	-0.13–0.11		-0.02	-0.16–0.12		0.09	-0.05–0.24	0.011
Neck pain (1–4)	1.90 (0.86)		1.87 (0.85)		1.95 (0.91)		0.05	-0.06–0.17		-0.04	-0.17–0.07		-0.06	-0.21–0.09		-0.07	-0.23–0.08	<0.001
Pain in shoulder and upper arm (1–4)	1.89 (0.88)		1.94 (0.89)		1.97 (0.95)		0.05	-0.07–0.18		-0.01	-0.13–0.12		-0.12	-0.28–0.03		-0.14	-0.31–0.01	<0.001
Back pain (1–4)	1.98 (0.92)		1.98 (0.84)		2.02 (0.89)		-0.02	-0.15–0.11		-0.12	-0.25–0.01		-0.09	-0.25–0.06		0.02	-0.13–0.18	0.009
Pain in hands, wrist or lower arm (1–4)	1.56 (0.81)		1.54 (0.81)		1.64 (0.88)		0.07	-0.05–0.20		0.05	-0.07–0.17		-0.01	-0.15–0.15		-0.02	-0.18–0.13	0.015
Pain in lower extremities (1–4)	1.95 (0.92)		1.93 (0.90)		1.85 (0.90)		0.01	-0.11–0.14		-0.10	-0.23–0.03		-0.03	-0.20–0.12		0.10	-0.05–0.27	<0.001

^a^ The municipal cluster – values below 0.001 shown as <0.001. ^b^ Higher rating indicates better self-reported health.

For physician-certified sick leave (table 4), there was a pattern of fewer sick leave days and periods due to musculoskeletal diagnoses and more sick leave days and periods due to psychological diagnoses after the interventions for both inspection and guidance workshops. However, none of these were statistically significant.

**Table 4:** Mixed negative binomial regression analysing the effect of the interventions on total number of days of sick leave and total number of sick leave periods for selected diagnoses groups. [IRR=incidence rate ratio; CI=confidence intervals]

		Total number of days of sick leave			Total number of sick leave periods	
		IRR	95% CI		IRR	95% CI
Musculoskeletal and psychological diagnoses ^a^						
	Inspection	0.89	0.50–1.59		0.93	0.72–1.21
	Guidance-through-workshop	0.98	0.54–1.76		1.05	0.81–1.36
Musculoskeletal diagnoses ^a^						
	Inspection	0.82	0.42–1.60		0.86	0.63–1.16
	Guidance-through-workshop	0.94	0.47–1.86		0.95	0.70–1.29
Psychological diagnoses ^a^						
	Inspection	1.08	0.35–3.31		1.13	0.68–1.85
	Guidance-through-workshop	1.16	0.36–3.69		1.32	0.80–2.18

^a^ Adjusted for outcome baseline values and percentage of full-time equivalent employment.

## Discussion

This study aimed to determine the effects of labor inspections and a guidance workshop intervention on self-reported health complaints and physician-certified sick leave due to musculoskeletal and psychological diagnoses of employees in home-care services. While there was a pattern of decrease in sickness absences due to musculoskeletal diagnoses and an increase in sickness absences due to psychological diagnoses in the intervention groups, we found no statistically significant effect of either interventions on physician-certified sick leave, or any of the self-reported health measures.

The EAVH project hypothesized that inspection and guidance would increase compliance with OSH legislation and regulations, which in turn would lead to improved psychosocial and ergonomic working condition and prevent employee ill-health and sickness absence. A previous study in the EAVH project found no effect of either inspection or guidance on a wide array of psychosocial and mechanical work factors (25), several of which have been linked to mental and musculoskeletal health (2–4, 8, 9). Given this lack of effect on work factors, one would expect limited potential of the two interventions to influence employee health and rates of sickness absence. Work factors other than those covered in Finnanger Garshol et al (25) could potentially influence sickness absence and employee health, and the interventions could have influenced how employers followed-up employee sickness absences. As such, unobserved factors could potentially explain some of the patterns seen regarding changes in physician certified sick leaves with a decrease in musculoskeletal-related sick leave and an increase in psychology-related sick leave. However, with self-reported health measures for mental distress and musculoskeletal complaints showing no similarly clear patterns, and with the patterns themselves not being statistically significant, it is difficult to make any inferences on potential causes for these patterns.

Organizational interventions are complex to develop and implement and challenging to evaluate (32). Two important factors for a successful intervention are: (i) the target audience being aware that there are issues that should be addressed and (ii) the content of the intervention being perceived as effective in addressing these issues (33, 34). The process evaluation of the EAVH project found that both interventions were implemented according to the protocol and that participants reported that the two interventions were both useful and educational (35). In addition, when asked whether they had plans to implement or had implemented changes in the work environment after the interventions, managers in the inspection intervention group were more likely to report having implemented or having plans to implement changes than managers in the control group (35). This indicates that the participants perceived that they had problems that needed to be rectified, and they found that the content of the interventions could be helpful in addressing these problems. As such, there is no evidence suggesting that the lack of substantial effects stems from a failure in the implementation of the interventions. However, we have little information on exactly what types of changes were implemented after the interventions and how outside circumstances affected the implementation of changes. As such we have no information on how the advent of COVID-19 impacted the study. Other studies have reported increased workload and working hours in the general healthcare services during the pandemic (36). In the home-care services, staff reported increased psychosocial strain during COVID-19, while managers reporting having less time for measures to improve employee wellbeing because of the pandemic (37). This suggests that COVID-19 might have attenuated the effects of the interventions both through increased load on staff and less time for managers to implement changes to the work environment.

The potential complexity of addressing work factors, health and sickness absence may also explain the lack of observable effects. The causes of ill-health and sickness absence are multifactorial, and while work factors account for a significant proportion (8, 9), many cases are attributable to causes and events outside of work (38). The causes may also vary within and between different work environments, with different work factors taking primacy. This is further illustrated by workplace interventions targeting musculoskeletal and psychological disorders exhibiting a large degree of heterogeneity regarding intervention components, settings, and population (39). Given this potential complexity, one-time inspections or single guidance workshops may not have had an adequate impact on the workplaces to influence employee health and the rate of sickness absence. More involved interventions such as labor inspections with subsequent follow-up guidance sessions or follow-up inspections may have had more of an impact. Alternatively, guidance workshops with several sessions over time to provide more guidance, feedback, and follow-up. However, all inspections and guidance by the NLIA are based on and limited by legislation, and the current rules and regulations may not be clear or defined enough. Weissbrodt & Giauque (40) highlighted that research within the field of labor inspections and psychosocial risk recommends better regulation and more specific legal requirements. This could potentially better inform enterprises of their duties, facilitate labor inspections, and in turn lead to more substantial changes in the work environment.

The present findings of the EAVH project are similar to those of Weissbrodt et al (41) who found that inspections primarily led to increased awareness of and competence in psychosocial issues and, to a lesser extent, any implementation of specific measures. Furthermore, they observed no effect on general working conditions. As such, based on the available research, the effects of regulatory tools are evident in more tangible areas of OSH, notably in reducing injuries (21, 22), while for psychosocial factors, which are more intangible, the effects of regulatory tools are unclear. Common measures to prevent accidents and injuries, such as implementing physical barriers, for example guardrails and protective clothing, exemplify this tangibility. Such measures, or the lack thereof, are more easily observed during inspections. Measures to prevent unsafe behaviors or psychosocial risk factors often includes relational or organizational components, such as addressing role conflict, changes in decision latitude or the distribution of job tasks, which are less readily observable, more complex, and require closer inspection and monitoring (42). Furthermore, while the standards and limits for physical and chemical exposures are set numbers, there are no such limits for psychosocial work factors. While such limits might be unfeasible in practice, legislation and regulations could enshrine some OHS requirements, such as requiring plans to prevent specific psychosocial risk factors, for example role conflict or high job demands.

### Strengths and limitations

The main strength of this study is its cluster randomized controlled design, which allows for inferences of cause-and-effect relationships. The use of registry data on certified sick leave ensured no recall bias and no loss of information due to dropout for this outcome. We based our data collection on standardized, validated measures to reduce measurement error. One limitation is the potential for self-selection bias in the study, as we have very limited information on those who declined to participate. Another limitation is the lower number of respondents compared to our initial estimates and goal from the study protocol (24), together with subsequent attrition. One potential reason for participant attrition could be the high levels of sickness absences and turnover in general in the home-care sector (12). The lower response rate and subsequent attrition could have introduced biases in the data, and those who stopped responding were generally younger, with less education and a lower mean employment percentage, and were more often in the “other healthcare staff” category. However, the differences were small, and the between-group distribution remained similar to that at baseline throughout the study period (25). Similar differences were observed among those who did not consent to the use of registry data. However, among those who consented, similarly to the main sample, there were no demographic between-group differences except for the employment percentage. The relatively low number of sickness absence cases due to the diagnoses of interest, that is musculoskeletal and psychological diagnoses, in the study population precluded any meaningful stratified analyses or analyses on separate diagnoses, indicating that only the category-level analyses were feasible. The participants were predominantly women; however, this reflects the current gender distribution in home-care services (17). We believe that these findings can be generalized to similar settings in the health and social care sectors, particularly in countries with similar legislation and regulations.

### Implications for practice and future research

The results suggest a need to further develop the content of regulatory tools to better address risk factors to occupational health in practice, for example through clearer and more defined regulations. The findings are in accordance with a previously noted lack of effect of regulatory tools on psychosocial and mechanical work factors (25), further suggesting a need for future studies on how regulatory tools can influence the work environment and prevent ill-health and subsequent sickness absence. Future research should also aim to further elucidate the effects of regulatory tools, for example using other methods and in different sectors.

### Concluding remarks

The present study found no statistically significant effects of labor inspections and guidance-through-workshops on self-reported health outcomes and physician-certified sick leave due to musculoskeletal or psychological diagnoses. The results should be interpreted with caution given the low study response rate and subsequent attrition on self-report measures, and in the context of the COVID-19 pandemic. Future studies, in various industries, should further elucidate whether regulatory tools influence employee health and sick leave due to musculoskeletal and mental disorders. Attention should also be given to how such regulatory tools and their content can be further developed to prevent sickness absence and employee ill health.

## Data Availability

Data will be available three years after project completion. Data access request will be reviewed by NSD - Norwegian Centre for Research Data/Sikt - Norwegian Agency for Shared Services in Education and Research.

## Collaborators

The EAVH project is a collaboration between the NLIA and STAMI. NLIA was responsible for providing the interventions and STAMI was responsible for the trial design, randomization, data collection, statistical analysis, data interpretation and drafting of the present manuscript.
